# Nitric Oxide Synthase Dysfunction Contributes to Impaired Cerebroarteriolar Reactivity in Experimental Cerebral Malaria

**DOI:** 10.1371/journal.ppat.1003444

**Published:** 2013-06-20

**Authors:** Peng Kai Ong, Benoît Melchior, Yuri C. Martins, Anthony Hofer, Pamela Orjuela-Sánchez, Pedro Cabrales, Graziela M. Zanini, John A. Frangos, Leonardo J. M. Carvalho

**Affiliations:** 1 Center for Malaria Research, La Jolla Bioengineering Institute, San Diego, California, United States of America; 2 Department of Bioengineering, University of California San Diego, San Diego, California, United States of America; 3 Parasitology Service, Evandro Chagas Clinical Research Institute, Fiocruz, Rio de Janeiro, Brazil; 4 Laboratory of Malaria Research, Oswaldo Cruz Institute, Fiocruz, Rio de Janeiro, Brazil; Faculdade de Medicina da Universidade de Lisboa, Portugal

## Abstract

Cerebrovascular dysfunction plays a key role in the pathogenesis of cerebral malaria. In experimental cerebral malaria (ECM) induced by Plasmodium berghei ANKA, cerebrovascular dysfunction characterized by vascular constriction, occlusion and damage results in impaired perfusion and reduced cerebral blood flow and oxygenation, and has been linked to low nitric oxide (NO) bioavailability. Here, we directly assessed cerebrovascular function in ECM using a novel cranial window method for intravital microscopy of the pial microcirculation and probed the role of NOS isoforms and phosphorylation patterns in the impaired vascular responses. We show that pial arteriolar responses to endothelial NOS (eNOS) and neuronal NOS (nNOS) agonists (Acetylcholine (ACh) and N-Methyl-D-Aspartate (NMDA)) were blunted in mice with ECM, and could be partially recovered by exogenous supplementation of tetrahydrobiopterin (BH4). Pial arterioles in non-ECM mice infected by Plasmodium berghei NK65 remained relatively responsive to the agonists and were not significantly affected by BH4 treatment. These findings, together with the observed blunting of NO production upon stimulation by the agonists, decrease in total NOS activity, augmentation of lipid peroxidation levels, upregulation of eNOS protein expression, and increase in eNOS and nNOS monomerization in the brain during ECM development strongly indicate a state of eNOS/nNOS uncoupling likely mediated by oxidative stress. Furthermore, the downregulation of Serine 1176 (S1176) phosphorylation of eNOS, which correlated with a decrease in cerebrovascular wall shear stress, implicates hemorheological disturbances in eNOS dysfunction in ECM. Finally, pial arterioles responded to superfusion with the NO donor, S-Nitroso-L-glutathione (GSNO), but with decreased intensity, indicating that not only NO production but also signaling is perturbed during ECM. Therefore, the pathological impairment of eNOS and nNOS functions contribute importantly to cerebrovascular dysfunction in ECM and the recovery of intrinsic functionality of NOS to increase NO bioavailability and restore vascular health represents a target for ECM treatment.

## Author Summary

Cerebrovascular dysfunction plays a key role in the pathogenesis of murine cerebral malaria (CM). Low nitric oxide (NO) bioavailability has been attributed as a cause of the dysfunction. However, it is unclear if the NO deficit could be inflicted by decreased NO production from its enzymatic sources (NO synthases (NOS)) in the brain. Here, we applied a novel cranial window method to elucidate the role of NOS in cerebrovascular dysfunction in murine CM. We found a state of endothelial NOS (eNOS) and neuronal NOS (nNOS) dysfunctions that are detrimental to cerebroarteriolar reactivity. Supplementation of tetrahydrobiopterin (BH4), a cofactor for NOS activation, ameliorates this effect. Supported by experimental findings on the downregulation of total NOS activity as well as the enhancement of oxidative stress, eNOS protein expression and conformational eNOS and nNOS inactivation in brains of mice with CM, we identified eNOS/nNOS uncoupling as a potential mechanism responsible for the NOS dysfunction. In addition, we discovered a downregulation of eNOS phosphorylation at its Serine residue (S1176) that can possibly decrease eNOS activity and exacerbate eNOS dysfunction. Our study provides new insights on the involvement of NOS in cerebrovascular dysfunction during CM and suggests NOS as a therapeutic target for CM.

## Introduction

Cerebral malaria (CM) is one of the most severe complications of malaria infection by Plasmodium falciparum that causes unacceptably high rates of mortality and morbidity, imposing devastating health and economic burdens especially in tropical countries [1]. The murine model of CM using C57BL/6 mice infected by Plasmodium berghei ANKA (PbA) is a well accepted animal model for studying CM as it shares many common pathological features with human CM [2]. The pros and cons of this model have been recently debated [3], [4], [5], [6]. Murine or experimental CM (ECM) is associated with a vasculopathy [7] which is distinctively characterized by widespread cerebral arteriolar vasoconstriction, intense microvascular inflammation and markedly reduced cerebral perfusion [8], suggesting that cerebrovascular function is severely compromised in ECM. Cerebrovascular function is central to blood flow regulation which in turn is critical for maintaining metabolic homeostasis required for proper neurological functions [9]. Since metabolic disturbances and neurological impairment are evident in ECM [10], [11], understanding the underlying mechanisms of cerebrovascular dysfunction will be imperative for providing mechanistic insights into pathogenesis of the disease.

Nitric oxide (NO) is an important physiological messenger in the brain that is implicated in the regulation of cerebrovascular tone, leukocyte adherence, platelets aggregation, synaptic transmission and cellular defense [12]. An emerging body of evidence has suggested that NO deficiency could delineate the pathogenesis of cerebrovascular dysfunction in ECM [13], [14]. Low cerebral NO levels could be triggered by pathological consequences from the malarial parasitic invasion of flowing erythrocytes in the blood stream [15] which include hypoargininemia, increased NO scavenging by cell-free hemoglobin, depleted nitrite levels and NO quenching by reactive oxygen species (ROS) [16], [17]. However, it is unclear if NO depletion could be directly linked to an intrinsic loss of NO production from its sources besides these extrinsic factors.

NO is produced by NO synthases (NOS) which in the brain can exist in three different isoforms, namely the neuronal NOS (Type I nNOS) and the endothelial NOS (Type III eNOS) that are constitutively expressed and the inducible NOS (Type II iNOS) that is induced by pathophysiological stimuli [18]. A fully functional NOS exists as homodimers and this stabilized form of the enzyme favors the biochemical production of NO from L-arginine (substrate) and oxygen (cosubstrate) with tetrahydrobiopterin (BH4) as cofactor [18]. eNOS, nNOS and to a much lesser extent iNOS functions are dependent on intracellular Ca^2+^, the increase of which facilitates binding of calmodulin (CaM) to the enzymes, stimulating the electron flow involved in NO production [18]. For eNOS and nNOS, their activation can also be induced by changes in phosphorylation states of their amino acid residues, specifically phosphorylation of Serine 1176 (S1176) (eNOS) [19] and Serine 1417 (S1417) (nNOS) [20] and dephosphorylation of Threonine 495 (T495) (eNOS) [19]. In particular, a major extracellular factor influencing eNOS(S1176) phosphorylation is the cerebrovascular wall shear stress, a mechanical stimulus that arises from blood flow in the brain vasculature [21].

Deficiency in BH4 is known to destabilize NOS dimers by promoting the uncoupling of redox activities in their monomers, resulting in ROS such as superoxide (O2^−^) being generated rather than NO [22]. Residual NO may also react with O2^−^ to form peroxynitrite (ONOO^−^) [12], a highly potent oxidant that is capable of exacerbating NOS uncoupling by enhancing oxidative degradation of BH4 [23]. Since the augmentation of oxidative stress is a well-recognized pathological outcome of malaria infection [24] which can potentially deplete BH4 [23], it is conceivable that in ECM, NOS uncoupling can contribute to low NO bioavailability, adversely affecting vascular tone in the brain. Although NOS uncoupling had been implicated in the pathogenesis of vascular disease states seen in coronary artery disease, atherosclerosis, ischemia/reperfusion injury, diabetes and hypertension [25], [26], [27], its role in ECM pathogenesis remains poorly understood. It is in consensus that NOS uncoupling in these diseases is intimately associated with BH4 deficiency induced by enhanced oxidative stress. Consistent with this notion, BH4 replenishment aimed at reversing NOS uncoupling and restoring vascular function has been successfully implemented in the treatment of these diseases in both experimental and clinical studies [25], [28].

In the present study, we investigated the role of NOS isoforms on cerebroarteriolar dysfunction in ECM and identified potential mechanisms responsible for the dysfunction. We revealed that eNOS and nNOS dysfunctions contribute importantly to the impairment of cerebroarteriolar responses in ECM which can be attributed to NOS uncoupling and downregulation of eNOS(S1176) phosphorylation. The findings provide a better understanding of the involvement of NOS in cerebrovascular dysfunction during ECM, helping to design rational therapeutic interventions to more effectively tackle CM.

## Results

### Parasitological and clinical parameters

Parasitemia, rectal temperature and motor score of mice used for superfusion, including those used in the assessment of pial vascular responses and nitrite/nitrate production, are shown in *Figs. S1A–C*. As expected, ECM mice developed hypothermia and were associated with rising parasitemia and lower motor score as compared to the uninfected mice. Similar findings were obtained for mice (Uninfected: 0.0%, 38.4±0.4°C & 22.8±0.4, n = 25; ECM: 11.9±4.6%, 30.3±1.9°C & 6.9±4.4, n = 24) used in all other studies (NOS activity, LPO (lipid hydroperoxide), western blot, and wall shear stress analyses).

### Arteriolar vessel diameters

**Figure 1:**
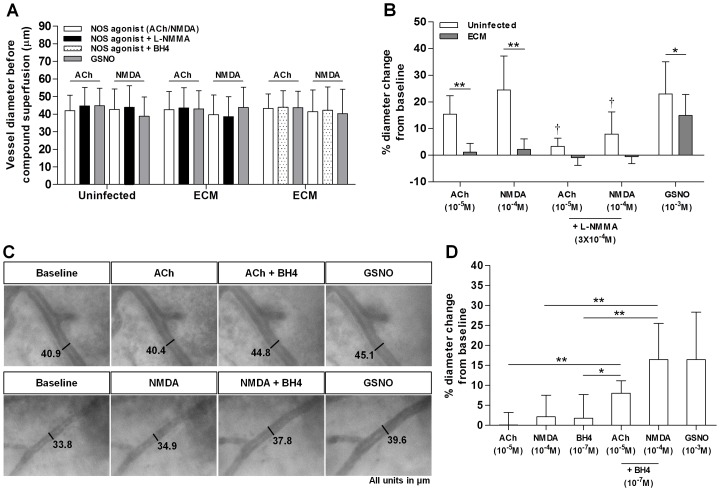
Pial arteriolar dilatory responses to ACh and NMDA are significantly impaired during ECM and can be partially restored by BH4 supplementation. **A**: Mean arteriolar vessel diameters before compound superfusion in all studies. **B**: Mean arteriolar responses from ACh and NMDA studies in uninfected and ECM mice. Number of vessels analyzed: ACh study: 17 uninfected and 15 ECM; NMDA study: 15 uninfected and 11 ECM. A total of 5 uninfected and 4 ECM mice were analyzed in each study. GSNO-induced responses were combined from both studies. ACh and NMDA-elicited arteriolar dilation in the uninfected mice were significantly decreased in the presence of L-NMMA. ACh, NMDA and GSNO-induced arteriolar dilation were significantly attenuated in the ECM mice as compared to the uninfected mice. **C**: Representative images of arterioles in ECM mice illustrating changes in their diameters after exposure to agonists (ACh & NMDA), BH4 with the agonist and GSNO. **D**: Mean arteriolar responses to application of the same compounds in (**C**). Number of vessels analyzed: 12 (ACh study) and 12 (NMDA study). A total of 4 ECM mice were examined for ACh/NMDA study. Responses elicited by BH4 alone or GSNO were combined from both ACh and NMDA studies. Mean dilatory responses to ACh and NMDA were significantly enhanced in the presence of BH4. *P<0.01, **P<0.0001. ^†^ *P*<0.0001; level of significance compared to treatments without L-NMMA.

To eliminate a possible effect of vessel size on the magnitude of arteriolar responses, we chose vessels of comparable diameters between all superfusion experiments. As shown in *Fig. 1A* [open bars], mean baseline arteriolar diameters of the vessels selected for the superfusion studies did not significantly vary among the different groups. We also ensured the return of arteriolar diameters to their baseline level before compound application during the superfusion experiment. Figure 1A illustrates that mean arteriolar diameters before the superfusion of compounds (NOS agonist+L-N^G^-monomethyl arginine (L-NMMA) [filled bars] or BH4 [hatched bars] and S-Nitroso-L-glutathione (GSNO) [grey bars]) were not significantly different from the baseline level in all superfusion groups.

### Arteriolar responses to agonists are blunted in ECM

An improved scheme of cranial window preparation for superfusion of the pial arterioles with test compounds was used to assess cerebroarteriolar function by the pial arteriolar reactivity during ECM. We exposed pial arterioles to classical eNOS and nNOS-dependent agonists (Acetylcholine (ACh) and N-Methyl-D-Aspartate (NMDA)) and a NOS-independent agonist in the form of an exogenous NO donor (GSNO), and examined possible changes in their diameter responses during ECM development. To verify the involvement of eNOS and nNOS in mediating the respective ACh and NMDA-induced arteriolar responses, we also make use of L-NMMA, a non-selective inhibitor of NOS. As shown in *Fig. 1B*, pial arterioles in the uninfected mice dilated prominently in response to ACh and NMDA applications and this effect was significantly attenuated by 78.4% (*P*<0.0001) and 67.6% (*P*<0.0001), respectively with L-NMMA treatment. This verified that ACh- and NMDA-induced pial arteriolar dilations were largely mediated by eNOS and nNOS, respectively. Pronounced mean arteriolar dilation was also found after GSNO application. On the other hand, mean dilatory responses to ACh and NMDA in the ECM mice were considerably decreased from those seen in the uninfected mice by 92.8% (*P*<0.0001) and 91.0% (*P*<0.0001), respectively. Any residual dilation was completely abolished with L-NMMA treatment. A significant attenuation (*P*<0.01) of mean GSNO-induced arteriolar dilation was also observed during ECM development but its magnitude (∼35%) was ∼2.6–2.7 folds less than those seen with ACh and NMDA. This implied that a NOS-independent mechanism can in part contribute to the impairment of ACh- and NMDA-elicited arteriolar responses in ECM but by a substantially lesser extent as opposed to relative contributions by eNOS- or nNOS-dependent mechanisms.

### BH4 treatment partially recovers impaired arteriolar responses in ECM

We further tested the hypothesis that BH4 deficiency, an effector of NOS uncoupling, accounts for the loss of ACh and NMDA-induced arteriolar dilation in ECM by challenging the possibility of BH4 supplementation in restoring these responses. Representative arterioles and changes in their diameters after applications of agonists (ACh & NMDA), agonist +BH4 and GSNO are shown in *Fig. 1C*. Notably, the application of the agonist together with BH4 was able to stimulate pronounced vasodilation (ACh: 9.5% & NMDA: 11.8%) that was not seen with the application of the agonist alone. Collectively, as presented in *Fig. 1D*, pial arterioles in ECM were not very responsive to ACh and NMDA applications. BH4 suffusion alone did not significantly alter mean baseline diameters whereas application of BH4 together with ACh or NMDA significantly enhanced dilatory responses to the agonist (*P*<0.0001) or BH4 alone (*P*<0.01 and *P*<0.0001, respectively). The resultant mean magnitudes of arteriolar dilation represented ∼52% and ∼67% restorations of respective mean ACh and NMDA-elicited responses that were observed in the uninfected mice, indicating that a deficiency of BH4 could in part contribute to the impairment of vascular responses during ECM. In addition, arterioles remained relatively responsive to GSNO, dilating with a mean magnitude of 16.5±11.9% which verified that vascular smooth muscle relaxation, though reduced, was active in the ECM mice. We also examined if the above observed changes in cerebroarteriolar responses were specific to ECM by repeating the superfusion protocol in C57BL/6 mice infected by Plasmodium berghei NK65 (PbNK65), a non-ECM-inducing parasite strain. Unlike the ECM mice, pial arterioles in these non-ECM-infected mice remained relatively responsive to both ACh and NMDA treatments (*Figs. S2A & S2B*). Their mean magnitudes of dilation were only partially blunted by 37% and 40%, respectively, from those seen in the uninfected controls, and were not significantly enhanced by BH4 supplementation. In addition, the extent of GSNO-mediated vasodilation in the PbNK65-infected mice was not significantly different from that in the uninfected mice, indicating that vascular smooth muscle activity remained intact in a non-ECM setting of malaria infection.

### NO generation by eNOS/nNOS agonists is blunted in ECM and can be partially restored by BH4 treatment

**Figure 2:**
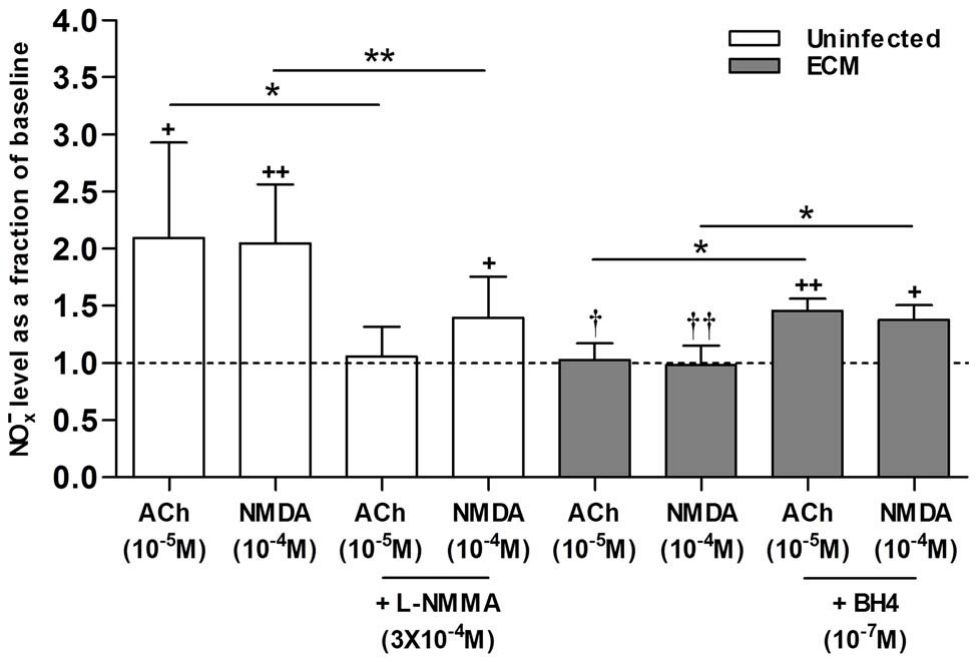
Production of NO metabolites upon application of ACh and NMDA is blunted in ECM and can be partially recovered by BH4 supplementation. Total NO metabolites 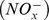, depicted by sum of nitrite and nitrate levels, are normalized to pre-treatment baseline (represented by dashed line). A total of 7 uninfected and 4 ECM mice were each analyzed in ACh or NMDA study. ACh/NMDA superfusion in uninfected mice produced a significant increase in 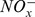 level from baseline (ACh: 1.9 µM→4.0 µM, NMDA: 2.0 µM→4.3 µM) which was significantly attenuated in the presence of L-NMMA. 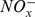 generation by ACh/NMDA application was significantly impaired in the ECM mice whereas BH4 supplementation partially restored 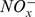 production by a significant extent. *P<0.05, **P<0.01. ^+^ *P*<0.05, ^++^ *P*<0.01; level of significance compared to baseline. ^†^ *P*<0.05, ^††^ *P*<0.01; level of significance compared to treatments in uninfected mice.

To verify that the observed changes in cerebroarteriolar responses in ECM were indeed elicited by changes in NO production by NOS, we assessed the extent of NO generation in response to the superfusion of different test compounds by the total amount of NO metabolites (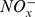; sum of nitrite and nitrate) in the superfusates. As shown in *Fig. 2*, ACh/NMDA superfusion induced a significant increase in 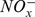 level from baseline (*P*<0.05 for ACh and *P*<0.01 for NMDA) in the uninfected mice. This change was significantly attenuated (*P*<0.05 for ACh and *P*<0.01 for NMDA) in the presence of L-NMMA, confirming that the observed pial vasodilatory responses produced by ACh/NMDA stimulation under physiological conditions were mediated by an increase in NO production. On the other hand, NO generation in response to the application of eNOS/nNOS agonists was found to be significantly blunted in ECM (*P*<0.05 for ACh and *P*<0.01 for NMDA) as 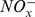 levels after ACh/NMDA superfusion in the ECM mice were not significantly different from baseline. However, with BH4 supplementation, the 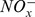 produced in response to the agonists was significantly enhanced (*P*<0.05 for both ACh and NMDA), corroborating that the partial recovery of the impaired ACh/NMDA-induced vasodilatory responses in ECM by BH4 was associated with restoration of NOS functionality to produce NO.

### Total NOS activity level is mitigated in ECM

**Figure 3:**
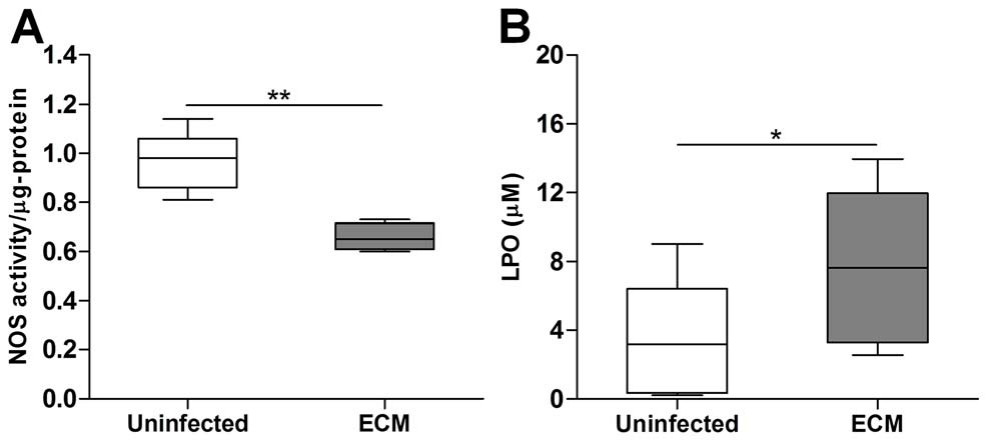
Cerebral NOS activity is mitigated while oxidative stress in the brain is augmented during ECM. **A & B**: Respective total cerebral NOS activity and LPO levels in uninfected and ECM mouse brains. Number of mice analyzed: NOS activity: 5 uninfected and 11 ECM; LPO: 7 uninfected and 11 ECM. Total NOS activity was normalized in relation to total protein concentration. Boxplots describe statistical distributions of NOS activity and LPO levels. Total NOS activity levels were significantly lower whereas LPO levels were significantly higher in ECM than uninfected mouse brains. *P<0.05, **P<0.001.

We verified NOS dysfunction in the brain during ECM using a NOS activity assay. In this assay, cofactors and substrates (including L-arginine) required for NOS activation were added to the brain lysate samples and total NOS activity level in each sample was quantified by the rate of L-citrulline formation normalized to the amount of protein in each sample. Figure 3A shows that mean total cerebral NOS activity level of ECM mice was significantly decreased (*P*<0.001) from that of uninfected mice, substantiating a decline in overall cerebral NOS function in ECM.

### Oxidative stress is enhanced in ECM

The level of lipid peroxidation in the brain measured using a LPO assay was used to provide a quantitative assessment of oxidative stress. We sought to examine if oxidative stress is enhanced in the brains of ECM mice as compared to uninfected mice. As shown in *Fig. 3B*, mean LPO level in the ECM mouse brains was significantly augmented (*P*<0.05) by approximately two folds as compared to the uninfected ones, demonstrating enhanced oxidative stress in the brain during ECM development.

### eNOS and iNOS protein expression are upregulated in ECM

**Figure 4:**
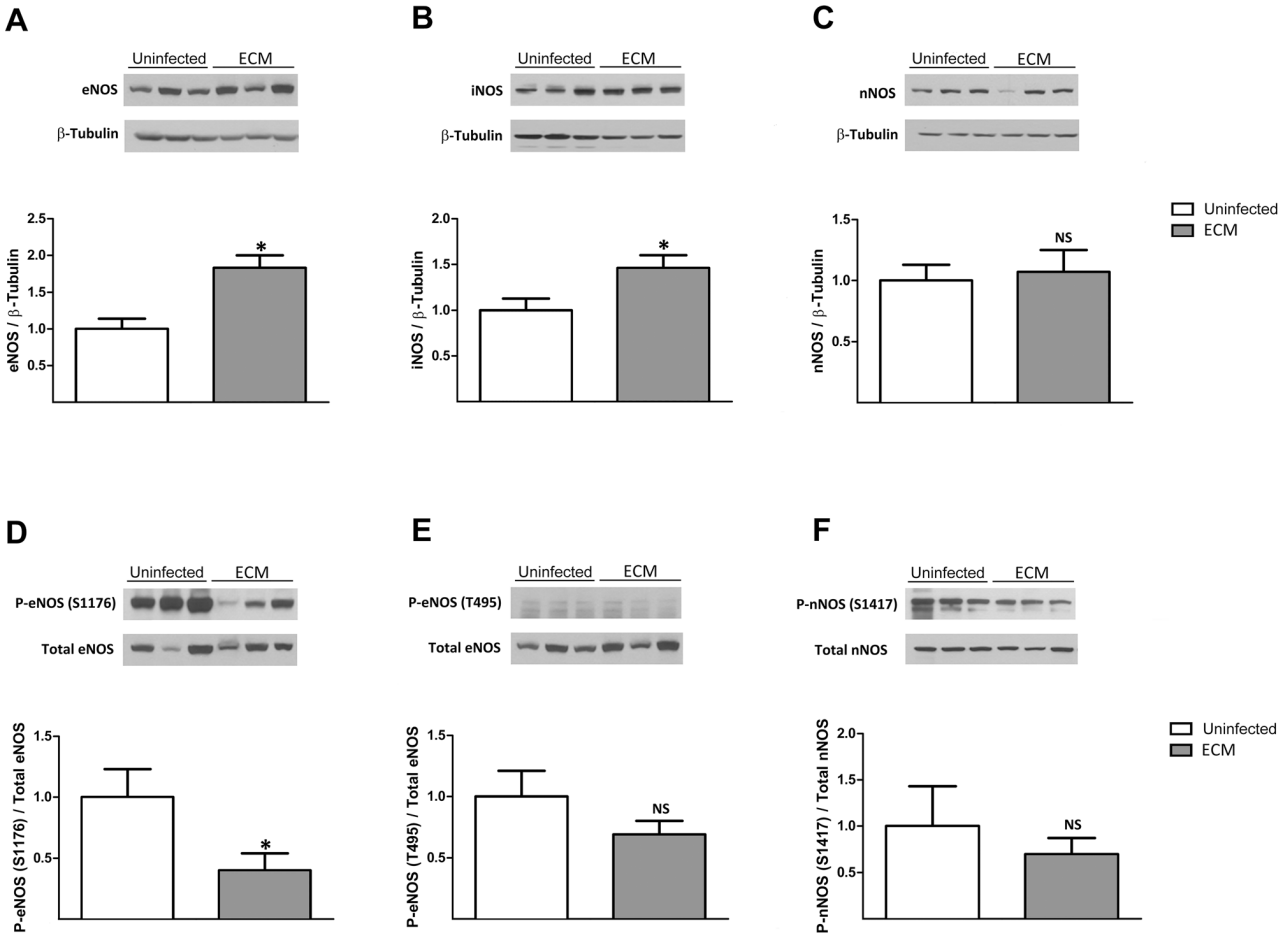
ECM increases total eNOS and iNOS expression levels and decreases eNOS(F176) phosphorylation. **A–C**: Bar graphs represent the western blot analyses of all NOS isoforms expression from the respective brain lysates as a ratio over total β-tubulin, normalized to the uninfected group. A representative blot for each condition is shown above the graph. eNOS and iNOS but not nNOS protein expression were significantly upregulated in ECM as compared to uninfected mouse brains. **D–F**: Immunoblots and bar graphs show ratio of phosphorylation level at each specific site to total amount of respective NOS. Phosphorylation level of eNOS(S1176) was significantly decreased in ECM but not those of eNOS(T495) and nNOS(S1417). A total of 7–10 uninfected and 7–10 ECM mouse brains were analyzed for each NOS isoform. *P<0.05, NS: non significant.

An increase in oxidative stress was previously found to induce an upregulation of renal and aorta eNOS and iNOS protein expression [29] which can induce NOS uncoupling through perturbation of the balance between BH4 and NOS bioavailability [30]. Hence, we examined if the protein expression of various NOS isoforms in the brain could be augmented under conditions of elevated oxidative stress in ECM. Western blot analysis of the denatured brain lysates indicated significantly increased amount of eNOS (*Fig. 4A*; *P*<0.05) and iNOS (*Fig. 4B*; *P*<0.05) but not nNOS isoforms (*Fig. 4C*) in ECM as compared to uninfected mouse brains.

### Phosphorylation of eNOS(S1176) is downregulated in ECM

To examine possible alterations of eNOS and nNOS activities in ECM via changes in their phosphorylation patterns, we probed brain protein lysates with primary antibodies specifically against the phosphorylated S1176 and the inhibitory T495 sites of mouse eNOS (*Figs. 4D & 4E*) and the phosphorylated S1417 site of mouse nNOS (*Fig. 4F*). Phosphorylation of S1176 of eNOS was significantly decreased (*P*<0.05) in ECM while no significant difference was observed between uninfected and ECM mice in cases of phosphorylation of eNOS(T495) and nNOS(S1417). It is noteworthy that although the total amount of eNOS almost doubled (shown above in *Fig. 4A*), the proportion of phosphorylated eNOS(1176) decreased by more than half and therefore the total levels of phosphorylated eNOS(S1176) remained lower in the ECM mice. Indeed, when normalized to β-tubulin, a 41% decrease (0.56±0.17→0.33±0.14) in total amount of phosphorylated eNOS(S1176) from uninfected controls was observed in ECM.

### Monomerization of eNOS and nNOS is enhanced in ECM

**Figure 5:**
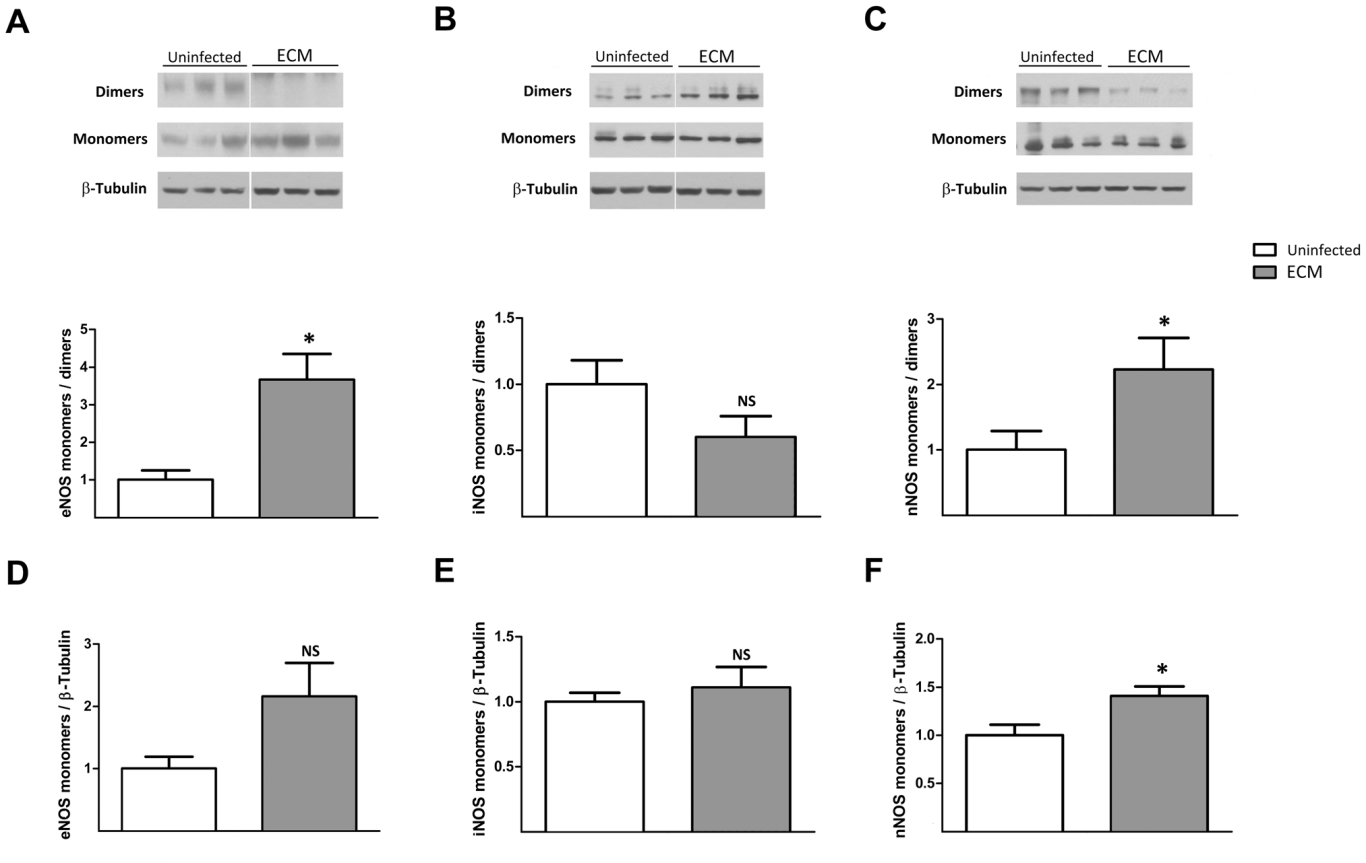
Monomerization of eNOS and nNOS is augmented in ECM. **A–C**: Non-denatured and non-boiled brain lysates from ECM and uninfected mice were probed for respective NOS isoforms. Monomer to dimer ratios are shown by bar graphs. Representative blots for each condition are shown above. Ratios of ECM brains were significantly increased from those of uninfected brains for eNOS and nNOS but not iNOS. **D–F**: Bar graphs represent ratio of amount of each NOS monomeric form to total amount of β-tubulin. Though not statistically significant (*P* = 0.059), amount of eNOS monomers increased by approximately two folds in ECM. Significantly elevated amount of nNOS but not iNOS monomers were also observed during ECM development. A total of 9–10 uninfected and 8–9 ECM mouse brains were analyzed for each NOS isoform. *P<0.05, NS: non significant.

Functional NOS dimers can become unstable and dissociate into their nonfunctional monomeric forms under conditions of enhanced oxidative stress [31], [32]. We asked if enhanced conformational inactivation of NOS isoforms by monomerization could contribute to NOS dysfunction in the brain during ECM development. The extent of NOS monomerization was analyzed in terms of the ratio between its monomeric and dimeric forms as well as the total amount of monomers. Undenatured brain lysates used to monitor the presence of SDS-resistant NOS dimers were run on a low temperature SDS-page for western blot analysis. In the case of eNOS, a significant increase in the ratio of monomers over dimers (*Fig. 5A*; *P*<0.05) was observed. Although there was an almost two-fold increase in its monomeric form (*Fig. 5D*), the change was not statistically significant (*P* value = 0.059). However, this observed increase in total monomeric form of eNOS, together with the significant augmentation of the monomers over dimers ratio, strongly indicated an enhanced eNOS monomerization in ECM. Furthermore, significant increases in both the ratio of monomers over dimers and the amount of monomeric forms of nNOS (*Figs. 5C & 5F*; *P*<0.05 & *P*<0.05) but not iNOS (*Figs. 5B & 5E*) were found in ECM as compared to uninfected mouse brains, supporting an augmentation of nNOS but not iNOS monomerization in ECM.

### Wall shear stress in microvessels is attenuated during ECM

**Figure 6:**
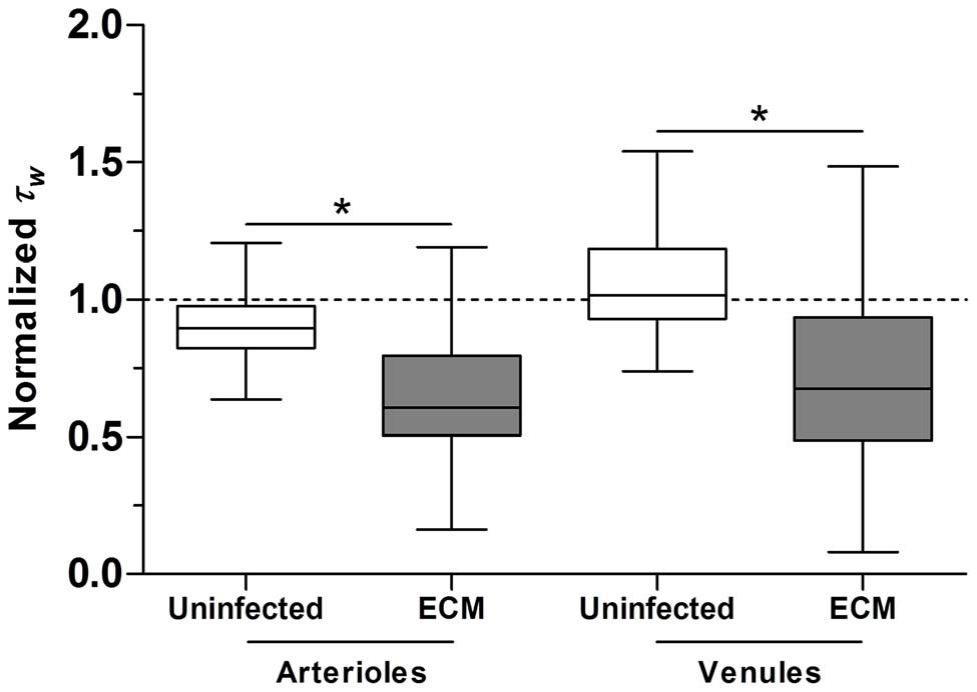
Wall shear stress in cerebral microvessels is significantly attenuated during ECM. Wall shear stress (*τ_w_*) on day 6 normalized to day 0 baseline levels (represented by dashed line) in arterioles and venules of uninfected and ECM mice. Number of vessels analyzed: uninfected: 36 arterioles and 48 venules; infected: 40 arterioles and 40 venules. A total of 9 uninfected and 8 ECM mice were analyzed. Normalized *τ_w_* was significantly lower in both arterioles and venules of ECM mice as compared to uninfected mice. Boxplot represents statistical distribution of *τ_w_* values. *P<0.0001.

Pathological alterations in the hemorheological properties of cerebral blood flow such as hematocrit, vessel diameter and flow velocity during malaria infection [13] could result in the modulation of cerebrovascular wall shear stress. Since a decrease in eNOS phosphorylation at S1176 was found in ECM, we questioned if this effect could be triggered by a corresponding reduction of wall shear stress during ECM development. By using a closed cranial window for chronic imaging of the pial microcirculation [33], a time-course study was performed comparing wall shear stresses (in venules and arterioles) between day 0 and day 6 (after ECM development) of PbA-infection in mice. Uninfected mice used as controls were correspondingly examined on both days. In uninfected mice, mean wall shear stress levels on day 6 were moderately altered in arterioles (2.81 Pa (baseline)→2.53 Pa) and venules (1.94 Pa→1.99 Pa) from their day 0 baselines (*Fig. 6*). In contrast, mean wall shear stress levels in the ECM mice decreased substantially from day 0 baselines (arterioles: 2.76 Pa→1.75 Pa & venules: 1.95 Pa→1.33 Pa) and these changes were significantly more pronounced (*Fig. 6*; *P*<0.0001) as compared to those in the uninfected mice.

## Discussion

The present study unveiled novel insights on changes in the status of NOS functions that contributes to cerebrovascular dysfunction in ECM. Substantial blunting of eNOS- and nNOS-elicited pial arteriolar dilatory responses during ECM development would point to a loss of enzymatic eNOS and nNOS activities involved in NO production, reinforcing the notion of low NO bioavailability in ECM that compromises cerebrovascular function. Several lines of evidence lent strong support to NOS uncoupling as a potential mechanism contributing to the NOS dysfunction which includes a state of BH4 deficiency, blunting of NO generation upon stimulation by agonists, decrease in total NOS activity, increase in lipid peroxidation levels, upregulation of eNOS protein expression and enhancement of eNOS and nNOS monomerization. In the case of eNOS, its dysfunction could be aggravated by a downregulation of phosphorylation activity at S1176 and this effect could be induced by the decrease in cerebrovascular wall shear stress during ECM development.

In the presence of a sustained increase in intracellular Ca^2+^, the dimeric form of NOS facilitates interdomain electron flow between its monomers [18]. The transferred electrons, together with a second electron supplied by BH4, reduce and activate O_2_ which in turn oxidizes L-arginine to generate NO and L-citrulline [18]. However, in the absence of BH4 or L-arginine, O_2_ is reduced to O_2_^−^ but L-arginine oxidation fails to occur. Therefore, NOS catalytic activity is uncoupled [18] and potent ROS are produced instead of NO. Although direct assessment of BH4 bioavailability was not performed in the present study, the reversible partial recovery of impaired eNOS- and nNOS-mediated pial arteriolar responses after BH4 supplementation would imply its insufficiency in ECM. Moreover, findings on the depletion of glutathione in our previous study [14] together with an increase in lipid peroxidation levels in the brains of ECM mice demonstrated an augmentation of oxidative stress during ECM. Oxidative stress is known to be capable of promoting the degradation of BH4 to biopterin (BH2) which lacks cofactor activity or suppressing the action of dihydrofolate reductase involved in BH4 recycling from BH2 via the salvage pathway [34]. Therefore, it seems likely that oxidative stress may drive NOS uncoupling in ECM by diminishing BH4 bioavailability, forming a vicious positive feedback loop of enhanced oxidation stress, BH4 depletion and NOS uncoupling which intensifies the decrease in NO production and impairs NOS-mediated cerebroarteriolar reactivity. Hypoarginemia may also play a role in NOS uncoupling during ECM [17]. However in the case of eNOS, Bevers and coworkers [35] reported that BH4 but not L-arginine supplementation was capable of ameliorating eNOS uncoupling in microvascular endothelial cells overexpressing the enzyme. In view of eNOS overexpression in ECM, it is probable that BH4 rather than L-arginine deficiency is the key factor contributing to eNOS uncoupling during ECM. This may not be surprising since eNOS uncoupling leading to NO deficiency and vascular dysfunction is a common pathological feature of many diseases associated with oxidative stress-induced BH4 deficiency [27], [28], [31], [34].

The enhancement of oxidative stress as a result of NOS uncoupling could exacerbate NOS dysfunction by destabilizing the quaternary structure of the NOS dimers, causing them to dissociate into their nonfunctional monomeric forms [31], [32]. In the present study, increased levels of eNOS and nNOS monomerization indicate that enhanced conformational inactivation of eNOS and nNOS under conditions of elevated oxidation stress in the brain could contribute to NOS dysfunction in ECM.

The significant upregulation of total eNOS protein expression in the brain during ECM could be triggered by an increase in ROS activity under conditions of low NO bioavailability [29]. Similarly, a concomitant augmentation of eNOS protein and NADPH oxidases, a major source of ROS, had been identified in many vascular diseases and is recognized as a potential mechanism that exacerbates their pathogenesis through enhancement of oxidative stress by eNOS uncoupling [36]. Increased eNOS protein expression accompanied by potentially enhanced NOS uncoupling in ECM due to a lack of BH4 could lead to the generation of more ROS which can aggravate NO depletion.

Excessive NO production by increased iNOS expression is known to play an immunological role in many neurodegenerative diseases and can contribute to massive vasodilation in sepsis [9]. Although total iNOS protein expression was similarly found to be significantly upregulated in ECM, its NO synthesizing capability may be impaired by NOS uncoupling mediated by BH4 depletion. However, unlike eNOS and nNOS, the lack of a substantial change in iNOS monomerization during ECM development would rule out enhanced conformational inactivation of iNOS as a possible cause for diminished NO production in ECM. This corroborates a likely scenario of low NO bioavailability in ECM stemming from the predominant decrease in NO synthesis by its dysfunctional counterparts (eNOS and nNOS). It is also conceivable that strong iNOS induction in inflammatory cells might contribute to excess consumption of L-arginine and hence to substrate shortage for eNOS and nNOS, in addition to generating peroxynitrite that would contribute to NOS uncoupling. Further studies, for instance using iNOS-deficient mice, are necessary to address these potential interactions between the different NOS isoforms in vascular dysfunction in ECM.

Unlike ECM, pial arteriolar responses to the application of eNOS/nNOS agonists were only partially attenuated from uninfected levels and were not significantly enhanced by BH4 in a non-ECM setting of murine malaria. These findings suggest that eNOS/nNOS dysfunction due to BH4 deficiency is more likely to mediate the impairments in pial arteriolar responses in ECM as opposed to non-ECM cases of malaria infection. Although some degree of eNOS/nNOS dysfunction may still exist in a non-ECM setting, it seems probable that majority of its associated blunted responses could be triggered by NOS-independent mechanisms that scavenge NO produced by NOS.

CD8^+^ T cells have been shown to be key effectors of ECM, and mice without CD8^+^ T cells fail to develop the disease [37], [38]. NOS inhibition has been shown to induce endothelial cell adhesion molecule expression and increase leukocyte adhesion [39], [40]. In our previous studies [13], [41], we showed that exogenous NO supplementation decreased vascular inflammation (leukocyte and platelet adherence and endothelial cell adhesion molecule expression) and damage (microhemorrhages and leakage). Therefore, in addition to contributing to vasoconstriction and impaired cerebrovascular responses, it is possible that NOS dysfunction may also contribute to vascular inflammation and possibly facilitate CD8^+^ T cell recruitment in ECM.

eNOS can also be activated independent of a sustained increase in intracellular Ca^2+^ through phosphorylation of S1176 [18] which can be evoked in the brain by wall shear stress exerted by blood flow on cerebrovascular endothelial cells lining the luminal vessel wall [42]. In this study, we provide evidence of a significant decrease in wall shear stress in cerebral microvessels during ECM which resulted from both the reduction of blood viscosity (*Fig. S3A*) due to anemia (decrease in mean *H_sys_*; Text S1) and the lowering of wall shear rate (*Fig. S3B*) by a predominant decrease of blood flow velocity over vessel diameter (Text S1). Our findings on the significant decrease in phosphorylated S1176 level in ECM lend support to the reduction of cerebrovascular wall shear stress by hemorheological changes as a potential mechanism synergistically contributing to the downregulation of eNOS activity during ECM. On the other hand, a phosphorylated state of T495 in unstimulated cells is known to deactivate eNOS by interfering with binding of CaM to the enzyme. Dephosphorylation of T495 increases eNOS activity and unlike S1176, this mechanism is less sensitive to changes in wall shear stress [21]. Instead, changes in T495 phosphorylation are generally associated with stimuli (e.g. bradykinin, histamine and Ca^2+^ ionophores) that elevate Ca^2+^ [19]. In the case of nNOS, phosphorylation at its S1417 is known to activate the enzyme and enhance the neuronal production of NO in the presence of Ca^2+^- CaM complex [20]. Levels of T495 and S1417 phosphorylation in ECM were not significantly different from those seen in the absence of infection, suggesting that these phosphorylation mechanisms are unlikely to mediate eNOS and nNOS dysfunctions in ECM.

Contradictory to the many beneficial effects of BH4 on ameliorating pathological deficits [43], [44], [45], our recent study [46] failed to prevent ECM by systemic BH4 supplementation which could be in part attributed to the rapid oxidation of BH4 in vivo to BH2 and failure to improve the BH4:BH2 ratios in tissues [27]. In addition, our findings revealed that BH4 supplementation in improving cerebroarteriolar responses in ECM was only effective when applied in conjunction with a NOS agonist. This suggested that not only reversing the NOS uncoupling but also restoring a possible pathological depletion of intrinsically produced NOS agonists are essential for tackling the cerebrovascular dysfunction in ECM.

The failure of BH4 to fully restore cerebroarteriolar dilatory responses would suggest that NOS uncoupling due to the cofactor deficiency is not entirely responsible for cerebrovascular dysfunction in ECM. Indeed, we observed a significant loss of pial arteriolar responses in ECM even with the application of a NOS independent agonist (GSNO). Therefore, additional mechanisms must be present that are deleterious to cerebrovascular function. One plausible mechanism would be the rapid quenching of the NO by massive amount of cell-free hemoglobin and ROS generated in ECM, which attenuates the effective amount of NO reaching and activating the effector vascular smooth muscle cells. Since biochemical insults in ECM can produce detrimental effects on cellular and tissue functions [24], another possibility would be the loss of smooth muscle activity by induced cellular death [47] or disruption of signaling pathways (e.g. decreased soluble guanylate cyclase (sGC) sensitivity to NO stimulation) leading to cyclic guanosine monophosphate (cGMP) downregulation [48]. It is also conceivable that NO-induced cerebroarteriolar dilation may be in part counteracted by the upregulated presence of potent vasoconstrictors such as endothelin-1 (ET-1) in ECM [49]. This can be further supported by a clinical study where the balance of vasoactive substances in the blood plasma of malaria patients was found to have shifted to one that favors vasoconstrictory (ET-1) over vasodilatory (C-type natriuretic peptide) effects [50].

Since low NO bioavailability and its associated vascular dysfunction are key factors contributing to ECM pathogenesis [13], [17], there is a compelling need for the development of new therapeutic approaches targeting the restoration of both NO bioavailability and cerebrovascular function for ECM treatment. Recently, adjunctive interventions that involve exogenous restoration of NO levels had been promising in improving microcirculatory function and decreasing overall incidence of ECM [13], [14]. Our current findings suggested that the use of BH4 with a NOS agonist could be a potential alternative to exogenous NO donors for ECM treatment by recovering the intrinsic functionality of NOS to produce NO and possibly prevent their undesired generation of potent ROS. These studies will demand the development of strategies to overcome BH4 oxidation occurring upon systemic delivery which has been proven by others to be the bottleneck targeting delivery to the brain [51]. Besides direct administration of BH4, it may be worthwhile to consider the modulation of endothelial GTPCH, a regulator of BH4 synthesis in vivo [52], to increase BH4 bioavailability.

## Materials and Methods

### Infection of mice

This study was carried out in strict accordance with the recommendations in the Guide for the Care and Use of Laboratory Animals of the National Institutes of Health. All experimental protocols were reviewed and approved by the Institutional Animal Care and Use Committee of La Jolla Bioengineering Institute (Permit Number: NO-CM003), and all efforts were made to minimize suffering. Six- to eight-week-old female C57BL/6 mice (Jackson Laboratories, Bar Harbor, ME) were intraperitoneally inoculated with 10^6^ PbA expressing the green fluorescent protein or PbNK65 (donations from the Malaria Research and Reference Reagent Resource Center - MR4, Manassas, VA; PbA-GFP: deposited by CJ Janse and AP Waters, MR4 number: MRA-865 and PbNK65: deposited by V Nussenzweig, MR4 number: MRA-268) for induction of ECM and non-ECM cases of malarial infection, respectively.

### Parasitological and clinical parameters

In PbA-infected mice, parasitemia was determined using flow cytometry by detecting and counting the number of parasitic cells (pRBCs) that expresses GFP in relation to 10000 red blood cells (RBCs) from a small blood sample (∼1 µl) obtained by a mouse tail end prick. In the case of mice infected by the non-fluorescent PbNK65, thin blood smears made from a drop of tail blood and stained with Giemsa were examined under a light microscope at ×1,000 magnification with an oil immersion lens (Nikon Eclipse E200; Nikon Instruments Inc, NY, USA). Parasitemia was then calculated by counting the number of pRBCs in at least 1,000 RBCs. Rectal temperature was measured with a thermocouple probe (Oakton Acorn; Oakton Instruments, IL, USA). Motor behavioral score was determined by a composite scoring system based on six motor behavior tests modified from the SHIRPA protocol [53]. ECM was defined as the presentation of one or more of the following clinical signs of neurological involvement: ataxia, limb paralysis, poor righting reflex, seizures, roll-over and coma.

### Window preparation for cranial superfusion

A new cranial window preparation scheme with improved stability was adopted here (Refer to Text S1). In brief, the scheme involved performing the major surgical procedures (skin removal and skull drilling) to create a skull bone flap in the healthy mice which were left to recover for approximately a week. Mice were then randomly assigned to the uninfected and infected groups. Animals in the uninfected group were left for another week before cranial window implantation. On the other hand, for animals in the infected group, window implantation was carried out on day 5–6 of infection after they had developed clinical signs of ECM with hypothermia and low motor behavioral score [8]. Window implantation was performed by first retracting the bone flap and subsequently assembling a prefabricated perfusion chamber to enable superfusion of superficial pial arterioles on the exposed brain cortical surface.

### Intravital microscopy and superfusion procedures

Uninfected and ECM mice with implanted cranial windows for superfusion were subjected to intravital microscopy. Pial arterioles were visualized through the cranial window and their vessel diameter responses were tested by sequential superfusion of the cortical surface with soluble test compounds. In the first series of superfusion experiment, responses to eNOS- and nNOS-dependent agonists (ACh (10^−5^M) and NMDA (10^−4^M)), the agonists with non-selective NOS inhibitor, L-NMMA (3×10^−4^M), and NOS-independent agonist (GSNO (10^−3^M)) were examined. In the event where arteriolar dilatory responses to ACh/NMDA were found to be impaired in the ECM mice, a second series of superfusion study was conducted in the sick animals using the agonist (ACh/NMDA), the agonist with BH4 (10^−7^M) and GSNO. To test if the vascular responses obtained with these compounds were specific to ECM, the superfusion protocol was repeated in PbNK65-infected animals. For assessment of nitrite/nitrate production upon superfusion of the test compounds, the superfusion protocols in both uninfected and ECM mice were repeated with the compounds dissolved in pure aCSF. Detailed descriptions on the experimental setup and superfusion protocols are provided in Text S1.

### Nitrite/nitrate measurement

Nitrite and nitrate levels in superfusates were determined using an automated NO detector-HPLC system (ENO-20; Eicom, San Diego, CA, USA) according to the manufacturer's instructions described in Text S1.

### Brain sample preparation

Infected mice were euthanized on day 6 of infection after the animals had developed clinical signs of ECM. After euthanasia, brains were harvested and were immediately flash frozen in liquid nitrogen and stored at −80°C for subsequent analyses of NOS activity, LPO and protein expression levels. Brains from uninfected mice were used as controls.

### NOS activity assessment

NOS activity levels in the brain samples were analyzed using a NOS activity assay according to the manufacturer's instructions (Cayman's NOS Activity Assay Kit – Catalog no. 781001) described in Text S1. The protein concentration of each brain lysate was also determined using a Bradford assay (Cayman's Protein Determination Kit – Catalog no. 704002). NOS activity was quantified by reaction rate of citrulline formation (pmoles citrulline formed per hour) normalized to the amount of protein in the lysate.

### LPO assessment

LPO levels in the brain samples were analyzed by a LPO assay kit according to manufacturer's instructions (Cayman's LPO Assay Kit – Catalog no. 705003) described in Text S1.

### Western blot analysis

Brains were homogenized on ice with an Ultra-Turrax (Ika, Werke) in RIPA lysis buffer and processed as previously described [54]. To preserve dimer forms of NOS, samples were loaded on prechilled 4–12% gradient gels (Invitrogen) and ran at slow voltage in the cold. Fractions of the LDS resuspended lysates were reduced and boiled when used to detect total amounts of each NOS isoform and its respective phosphorylated form(s), where applicable. After transferring onto polyvinylidene difluoride membranes and blocking and incubating with specific primary antibodies as detailed in Text S1, bound antibodies were detected with horseradish peroxydase-conjugated secondary antibodies (Cell Signaling Technology). Band intensity (mean optical density integrated for the band area) was quantified on unsaturated X-ray films and with ImageJ. Levels of total NOS expression and NOS monomers were presented as a ratio over β-tubulin content and normalized to the uninfected group.

### Wall shear stress calculation

By using the closed cranial window preparation for chronic imaging of brain microhemodynamics as described in our previous study [33], vessel diameter (*D*) and centerline flow velocity (*V_c_*) were determined in arterioles and venules (baseline vessel diameter = 19–65 µm) of both uninfected and ECM mice on day 0 and day 6 of infection. Systemic hematocrits (*H_sys_*) of the animals were obtained as described previously [13]. Wall shear stress (*τ_w_*) was calculated by the product of apparent blood viscosity (*μ_app_*) and wall shear rate (*γ_w_*) in the vessel. *μ_app_* which is a function of *H_sys_* was estimated based on an *in vivo* empirical relationship (parameters derived from the rat mesentery) proposed by Pries et al. [55] (Refer to Text S1). By assuming a parabolic flow velocity profile in the vessel, *γ_w_* was approximated by 4*V_c_*/*D* (Refer to Text S1). Wall shear stress values were normalized to their baseline levels on day 0.

### Statistical analyses

All statistical analyses were performed using a statistical software package (Prism 5.0, Graphpad). In all studies, unpaired or paired two-tailed student t-test was used to determine the statistical significance of the differences between uninfected and ECM groups or the effect of L-NMMA/BH4 treatment (in the case of arteriolar response and NO metabolites). All reported data were in mean ± standard deviation (SD) except for those from the western blot experiments which were in mean ± standard error of the mean (SEM). *P*<0.05 was considered statistically significant.

## Supporting Information

Figure S1 **Parasitological and clinical parameters of mice used for superfusion.** **A–C**: Parasitemia, temperature and motor score of uninfected and ECM mice. Open and closed circles represent individual uninfected and ECM mice, respectively.
(TIF)

Figure S2 **Pial arteriolar dilatory responses to ACh and NMDA are not significantly modulated by BH4 supplementation in PbNK65-infected mice.** **A**. Representative images of arterioles in PbNK65-infected mice showing changes in their diameters after exposure to agonists (ACh & NMDA), BH4 with the agonist and GSNO. **B**. Mean arteriolar responses to application of the same compounds in (**A**). Mean diameter and number (n) of vessels analyzed: 48.2±10.1 µm, n = 13 (ACh study) and 42.1±11.1 µm, n = 16 (NMDA study). A total of 3 mice were examined for ACh/NMDA study. Responses elicited by GSNO were combined from both ACh and NMDA studies. Superfusion of test compounds were performed in these mice between days 6 and 8 of infection when their parasitemia reached levels (10.9±2.1%) comparable to those in the PbA-infected mice. As expected, all of the mice did not develop any clinical signs of ECM at the time of investigation, and their temperatures (38.2±0.4°C) and motor scores (21.8±0.8) were also not significantly different from those in uninfected animals. Dilatory responses to ACh (9.7±5.9%) and NMDA (16.7±9.0%) were not significantly affected in the presence of BH4 and vessels remained highly responsive to GSNO, dilating by 22.1±9.6%.
(TIF)

Figure S3 **Apparent blood viscosity and wall shear rate in cerebral microvessels are attenuated during ECM.** **A & B**: Respective apparent blood viscosity (*μ_app_*) and wall shear rate (*γ_w_*) levels on day 6 normalized to day 0 baselines (represented by dashed line) in arterioles and venules of uninfected and ECM mice. Number of vessels analyzed: uninfected: 36 arterioles and 48 venules; infected: 40 arterioles and 40 venules. A total of 9 uninfected and 8 ECM mice were analyzed. Boxplot represents statistical distribution of *τ_w_* values. *P<0.0001. In the uninfected mice, mean *μ_app_* was not significantly altered from baseline for both vessel types, whereas mean *γ_w_* was found to decrease by a moderate extent from baseline in arterioles (627 s^−1^ (baseline)→563 s^−1^, 0.90±0.12 of baseline) but not in venules (441 s^−1^→449 s^−1^, 1.04±0.16). On the contrary, substantial attenuations of both parameters from baseline levels (*μ_app_*, arterioles: 3.65cP→3.34cP, 0.92±0.11 & venules: 3.60cP→3.21cP, 0.89±0.07; *γ_w_*, arterioles: 616 s^−1^→427 s^−1^, 0.67±0.29 & venules: 445 s^−1^→341 s^−1^, 0.78±0.30) were generally observed for both vessel types in the ECM mice and the magnitudes of these changes were significantly greater than those seen in the uninfected mice.
(TIF)

Text S1 **Materials and Methods.**
(DOC)
