# Supplementary figures and images for: Nitric Oxide Synthase Dysfunction Contributes to Impaired Cerebroarteriolar Reactivity in Experimental Cerebral Malaria
Source: PLoS Pathog. 2013 Jun 20;9(6):e1003444. doi: 10.1371/journal.ppat.1003444 (PMC3688552; doi:10.1371/journal.ppat.1003444)

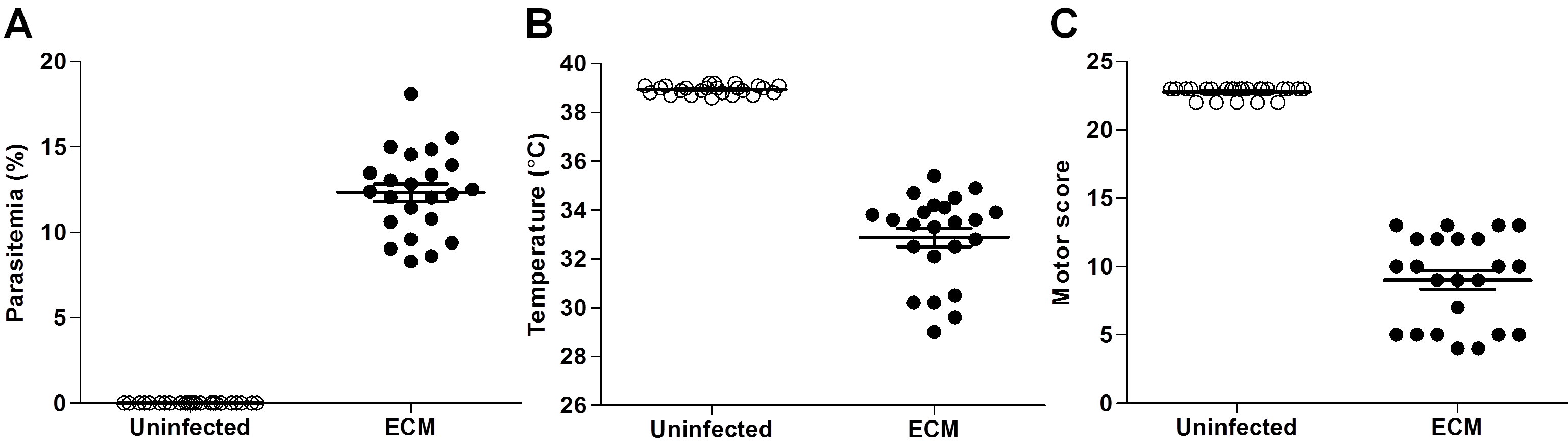

Supplement: Figure S1 — Parasitological and clinical parameters of mice used for superfusion. A–C: Parasitemia, temperature and motor score of uninfected and ECM mice. Open and closed circles represent individual uninfected and ECM mice, respectively. (TIF) [file ppat.1003444.s001.tif]

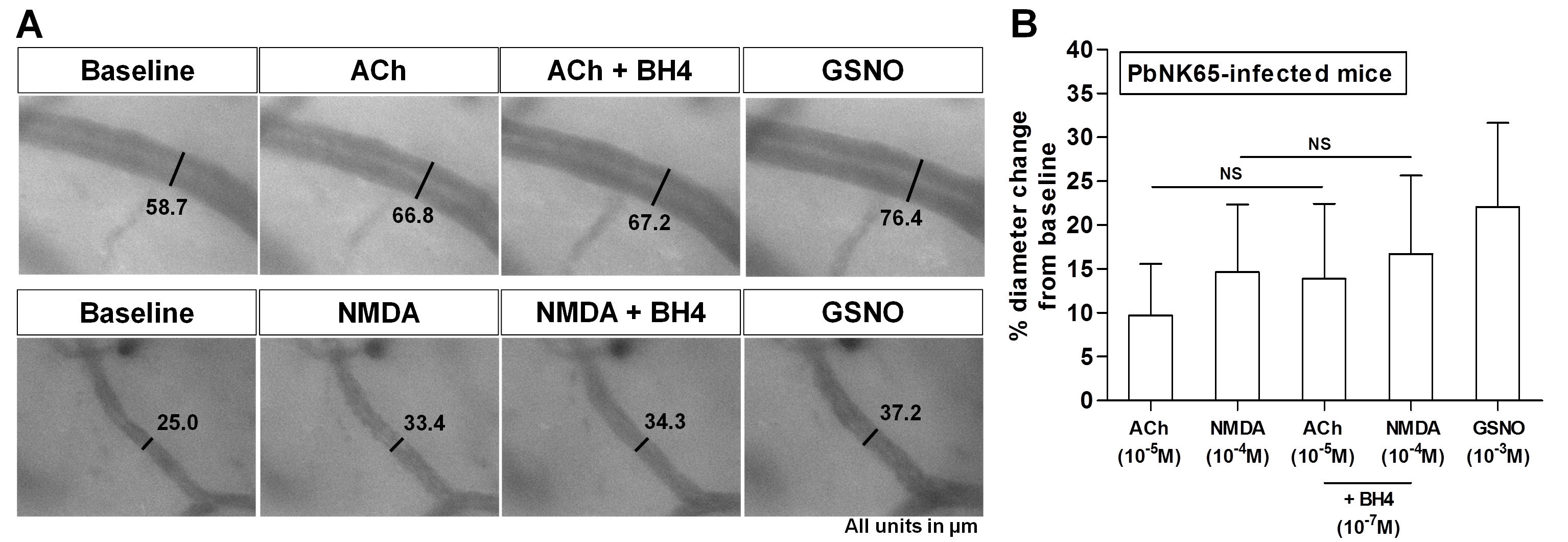

Supplement: Figure S2 — Pial arteriolar dilatory responses to ACh and NMDA are not significantly modulated by BH4 supplementation in PbNK65-infected mice. A. Representative images of arterioles in PbNK65-infected mice showing changes in their diameters after exposure to agonists (ACh & NMDA), BH4 with the agonist and GSNO. B. Mean arteriolar responses to application of the same compounds in (A). Mean diameter and number (n) of vessels analyzed: 48.2±10.1 µm, n = 13 (ACh study) and 42.1±11.1 µm, n = 16 (NMDA study). A total of 3 mice were examined for ACh/NMDA study. Responses elicited by GSNO were combined from both ACh and NMDA studies. Superfusion of test compounds were performed in these mice between days 6 and 8 of infection when their parasitemia reached levels (10.9±2.1%) comparable to those in the PbA-infected mice. As expected, all of the mice did not develop any clinical signs of ECM at the time of investigation, and their temperatures (38.2±0.4°C) and motor scores (21.8±0.8) were also not significantly different from those in uninfected animals. Dilatory responses to ACh (9.7±5.9%) and NMDA (16.7±9.0%) were not significantly affected in the presence of BH4 and vessels remained highly responsive to GSNO, dilating by 22.1±9.6%. (TIF) [file ppat.1003444.s002.tif]

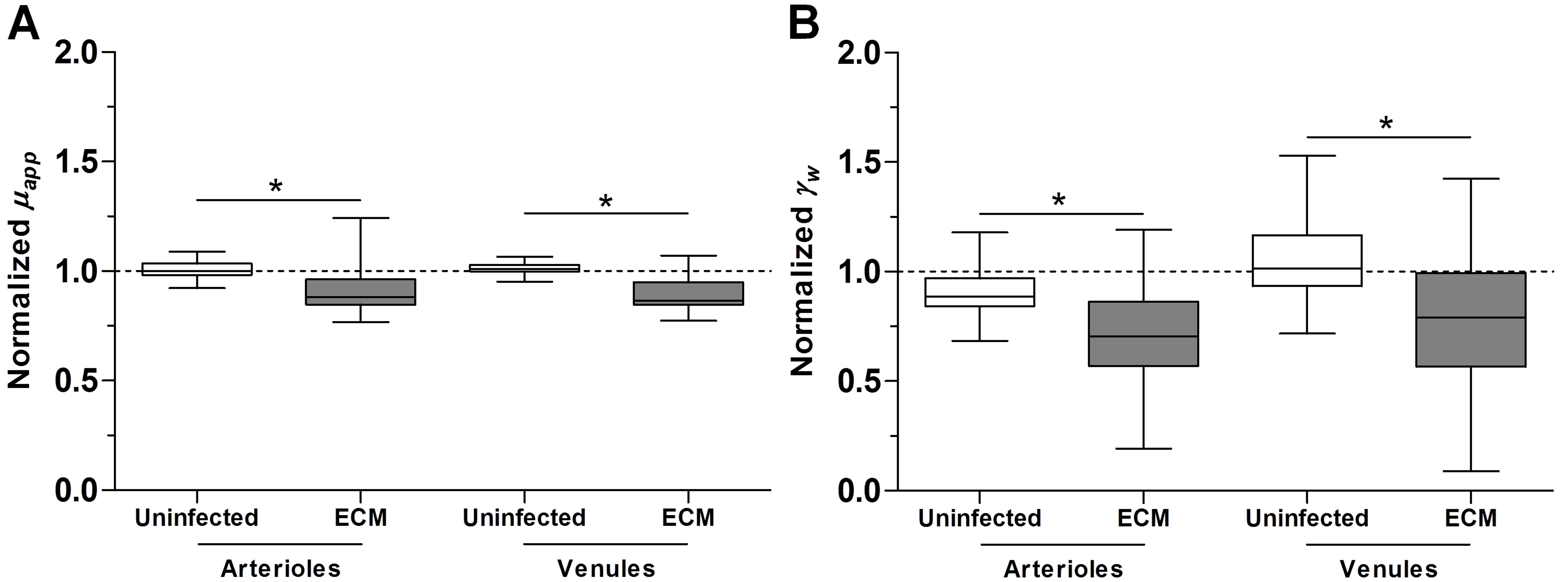

Supplement: Figure S3 — Apparent blood viscosity and wall shear rate in cerebral microvessels are attenuated during ECM. A & B: Respective apparent blood viscosity (μapp) and wall shear rate (γw) levels on day 6 normalized to day 0 baselines (represented by dashed line) in arterioles and venules of uninfected and ECM mice. Number of vessels analyzed: uninfected: 36 arterioles and 48 venules; infected: 40 arterioles and 40 venules. A total of 9 uninfected and 8 ECM mice were analyzed. Boxplot represents statistical distribution of τw values. *P<0.0001. In the uninfected mice, mean μapp was not significantly altered from baseline for both vessel types, whereas mean γw was found to decrease by a moderate extent from baseline in arterioles (627 s−1 (baseline)→563 s−1, 0.90±0.12 of baseline) but not in venules (441 s−1→449 s−1, 1.04±0.16). On the contrary, substantial attenuations of both parameters from baseline levels (μapp, arterioles: 3.65cP→3.34cP, 0.92±0.11 & venules: 3.60cP→3.21cP, 0.89±0.07; γw, arterioles: 616 s−1→427 s−1, 0.67±0.29 & venules: 445 s−1→341 s−1, 0.78±0.30) were generally observed for both vessel types in the ECM mice and the magnitudes of these changes were significantly greater than those seen in the uninfected mice. (TIF) [file ppat.1003444.s003.tif]
