# Supplementary material for: Nitric Oxide Synthase Dysfunction Contributes to Impaired Cerebroarteriolar Reactivity in Experimental Cerebral Malaria
Source: PLoS Pathog. 2013 Jun 20;9(6):e1003444. doi: 10.1371/journal.ppat.1003444 (PMC3688552; doi:10.1371/journal.ppat.1003444)
Supplement: Text S1 — Materials and Methods. (DOC) [file ppat.1003444.s004.doc]

**Text S1. Materials and Methods**

**Cranial window preparation for superfusion.** Mice were anesthetized with intraperitoneal (i.p.) administration of ketamine (150mg/kg) and xylazine (10mg/kg). Dexamethasone (0.2mg/kg), carprofen (5mg/kg) and ampicillin (6mg/kg) were injected subcutaneously. Full sedation of the animal was ensured during the surgery by repeatedly testing the animal reflex responses to toe/tail pinches. The scheme consists of two key steps: a presurgical step which involves performing the major surgical procedures in the animal and allowing it to recover and a chamber assembly step whereby a prefabricated perfusion chamber is assembled to enable superfusion of its exposed brain cortical surface. We have shown that this new scheme maintains stability in arterial blood pressure and baseline arteriolar diameter during the superfusion procedures .

In the presurgical step (**day 0**), most of the surgical procedures follow those commonly used in the preparation of a craniotomy (~3 mm) until the point of drilling a circular groove in the right parietal bone of the skull to create a bone flap . However, instead of retracting the bone flap to expose the brain cortex, low-temperature melt bone wax was used to cover it and the surrounding skull area was filled with dental acrylic. On the day following recovery from anesthesia (**day 1**), the mice were given analgesic and antibiotic (carprofen and ampicillin) daily for five days and left for another three days for the drug effects to subside. Animals presenting signs of pain or discomfort were euthanized intraperitoneally by using euthasol (100mg/kg). This presurgical step was designed to minimize surgical trauma in the sick mice on the day of the experiment which helps to improve preparation stability. Mice were randomly assigned to the uninfected and infected groups (**day 8**). Animals in the infected group were innoculated with PbA and the chamber assembly step was carried out on day 5-6 of infection (**day 13-14**) after they had developed clinical signs of ECM with hypothermia and low motor behavioral score . On the other hand, animals in the uninfected group were left for another 5-6 days (**day 13-14**) before execution of the chamber assembly step. Therefore, the chamber assembly step was performed in both groups at the same time point after the presurgical procedure. This serves as a time control for arteriolar responses in the ECM animals to ensure that any alteration of response observed during ECM infection is not a time-dependent phenomenon that would occur even in the absence of infection.

The chamber assembly step was initiated by anesthetizing the mouse using isofluorane (4% for induction, 1-2% for maintenance). By securing the animal in a prone position under a stereotaxic frame, the bone wax was gently scraped off the skull surface which was then cleaned with several swipes of sterile saline. After which, the exposed bone flap previously created was carefully lifted off under a drop of sterile saline using a pair of forceps to reveal the underlying cortex. The dura mater was carefully incised and retracted. Any minor bleeding of the exposed cortical surface was quenched using gelfoam soaked previously in sterile artificial cerebrospinal fluid (aCSF) (Harvard Apparatus, Holliston, Massachusetts) which has an ionic composition resembling the brain CSF (150mM Na, 3.0mM K, 1.4mM Ca, 0.8mM Mg, 1.0mM P & 155mM Cl). A prefabricated chamber consisting of a ring structure (internal volume of ~10µl, equipped with inlet and outlet ports connected to PE-10 tubes and a third port for intracranial pressure monitoring) was glued (Vetbond, 3M, St. Paul, MN) to the skull with an orientation that positions the exposed cortex at the center of its internal opening. Dental acrylic was applied around the chamber to secure its fixation and to prevent leakage. The space inside the chamber was then completely filled with sterile aCSF through the inlet tube and the assembly step was completed by covering the chamber top with a glued-on cover glass.

**Intravital microscopy.** After the chamber assembly, the isofluorane supply was discontinued and α-chloralose (Sigma-Aldrich, St. Louis, MO) was infused i.p. with a loading dose of 30 mg/kg, followed by a maintenance dose of 25 mg/kg every hour for the remainder of the experiment . The mouse was transferred to an intravital microscopic stage (customized Leica-McBain, San Diego, CA) and its body temperature maintained by a heating pad. The exposed brain cortical surface was preconditioned with the aCSF by continuously flushing prewarmed aCSF (37°C and pH maintained at 7.35-7.45 by equilibration with a gas mixture of 6.5% O2, 10% CO2 and balance N2) through the chamber for 30 mins, at a flow rate of 10 µl/min with the help of a syringe pump (Harvard Apparatus pump 33, Holliston, Massachusetts). During this period, the pial microcirculation was visualized through the cranial window under a drop of water using a 20X water immersion objective (LUMPFL-WIR, numerical aperture 0.6, Olympus). 1-4 arterioles (baseline vessel diameter = 20-70 µm) for analyses were randomly selected and identified on the basis of diverging flow patterns in their downstream ramifications. Spatial mapping of their locations in the window was carried out to enable ease of locating them afterwards. Sequential superfusion of the cortical surface with test compounds dissolved in aCSF was carried out at a flow rate of 10 µl/min controlled by the syringe pump while brightfield visualization of the arterioles was simultaneously performed based on epiillumination. The height of outlet tube was adjusted such that intracranial pressure was maintained between 5 and 8 mmHg . All vessel diameter measurements were performed using an image shear device (Image Shear, Vista Electronics, San Diego, CA) with a spatial resolution of 0.21 µm/pixel and images for this purpose were captured by a low light analogue camera (COHU 4815, San Diego, CA) and displayed on a color monitor (PELCO, Clovis, CA). For documentation purposes, images (320 by 240 pixels) of all vessels were captured using a digital low-light high speed camera (Hamamatsu C9300-221, Japan) in conjunction with an image acquisition software (HCImage, Hamamatsu, Japan).

**Superfusion procedures.** Arteriolar dilatory responses to eNOS-dependent agonist (ACh (Tocris Bioscience, Minneapolis, MN)) and to NOS independent agonist (GSNO (Cayman Chemical, Ann Arbor, Michigan)) were examined in both uninfected (n = 5) and ECM (n = 4) mice. In addition, the involvement of NOS in mediating ACh-induced responses was also tested using the non-selective NOS inhibitor, L-NMMA (Cayman Chemical, Ann Arbor, Michigan). Arteriolar response was defined by the percentage change in vessel diameter after superfusion of the compound(s). The superfusion protocol was as follow: Arteriolar responses to 5 mins of ACh (10^-5^M) superfusion were first obtained. The arterioles were then suffused with L-NMMA (3X10^-4^M) for 30 mins and their responses to ACh were retested by the combined superfusion of both ACh and L-NMMA for another 5 mins. After washout of these compounds by aCSF (10-15 mins) and upon recovery of baseline vessel diameters, responses to 5 mins of GSNO (10^-3^M) superfusion were further determined. The above protocol was repeated with nNOS-dependent agonist NMDA (10^-4^M; Tocris Bioscience, Minneapolis, MN) instead of ACh in another group of uninfected (n = 5) and ECM (n = 4) mice.

In the event where arteriolar dilatory responses to ACh/NMDA were found to be impaired in the ECM mice, the possibility of BH4 treatment in restoring the responses was tested in a separate group of mice with ECM (n = 4). In this experiment, arteriolar responses to 5 mins of the ACh (10^-5^M)/ NMDA (10^-4^M) superfusion were first determined, following which the vessels were suffused with exogenous BH4 (10^-7^M; Cayman Chemical, Ann Arbor, Michigan) for 30 mins. The responses to the eNOS/ nNOS agonist were then reexamined by combined superfusion of the agonist and BH4 for 5 mins. Finally, responses to 5 mins of GSNO (10^-3^M) superfusion were tested after aCSF washout of the compounds. The specificity of these vascular responses to ECM was also tested in PbNK65-infected mice (n = 4 for ACh/NMDA study), a non-ECM murine model of malaria infection. The concentrations of agonists (ACh and NMDA) and cofactor (BH4) were chosen based on reported values in literature where they were respectively shown to be capable of eliciting prominent pial arteriolar dilation and reversing BH4-associated loss of pial arteriolar dilation . Preliminary observations also revealed that the steady-state dilatory responses to all agonists (ACh, NMDA and GSNO) typically occur within 3-5 mins after their introduction.

**Nitrite/nitrate determination.** Superfusion procedures were repeated in uninfected and ECM mice (n = 7 and 4, respectively for ACh/NMDA study) with test compounds dissolved in pure aCSF and superfusates were collected from the outlet port of the chamber for 1.5 min before the cessation of their treatment. Nitrite/nitrate concentrations were determined using an ENO-20 automated detector-HPLC system (Eicom, San Diego, CA, USA) with a sensitivity of 10 nM, according to the manufacturer's instructions. In brief, a small sample (10µl) for analysis was carried by a mobile phase (containing 10% methanol containing 0.15M NaCl-NH_4_Cl and 0.5g/l of 4Na-EDTA) through a reverse-phase separation column packed with polystyrene polymer where the nitrite was separated from the nitrate. Nitrite was mixed with a Griess reagent (containing 1.25% HCL with 5g/l sulfanilamide and 0.25g/lN-napthylethylenediamine, delivered at a rate of 0.1ml/min) in a reaction coil. The formation of red colored diazo compounds, which provides an indication of the nitrite level, was measured by absorbance at 540 nm using a visible detector. Nitrate was further reduced to nitrite in a reduction column packed with copper-plated cadmium filings and subsequently also reacted with the Griess reagent to form diazo compounds for detection. Detected signals were captured by an EPC-300 data processor (Eicom) and their waveforms displayed and analyzed by PowerChrom software (eDAQ; Colorado Springs, CO, USA), with nitrite and nitrate levels given by area under peaks with a retention time of 4.5 and 8 min, respectively from the injection of the sample. The setup (separation column, reduction column and reaction coil) was maintained at 35°C in a column oven.

**NOS activity assessment.** NOS activity levels in the brain samples were analyzed using a NOS activity assay according to the manufacturer's instructions (Cayman's NOS Activity Assay Kit – Catalog no. 781001). In brief, each brain sample was homogenized on ice using a dounce homogenizer in Homogenization Buffer. The homogenates were centrifuged at 15,000 x g for 5 mins at 4°C and the supernatants transferred to clean tubes. The protein concentration of each lysate was determined using a Bradford assay (Cayman’s Protein Determination Kit – Catalog no. 704002). NOS assays were performed using 5µl of lysate. Background formation of citrulline was determined by assaying each sample in the presence of 1 mM of the NOS inhibitor L-NNA. Cofactors for NOS were added to the reaction mixture. The final reaction mixture contained the following components: 3µM Tetrahydrobiopterin, 1 mM Magnesium acetate, 600 µM Calcium chloride, 0.1 µM Calmodulin, 1 µM β-NADPH, 1 µM Flavin adenine dinucleotide and 30 µM Arginine. Samples were incubated for 30 mins at room temperature, the reactions were stopped by the addition of Stop Buffer and samples were processed as described in the kit booklet. After processing, the eluates were transferred to scintillation vials for counting. Scintillation cocktail was added and radioactivity was measured using a Packard 1550 Tri-Carb Liquid Scintillation Analyzer. Citrulline formation was determined and reaction rate (pmoles citrulline formed per hour) was normalized to the amount of lysate used in the assay.

**LPO assessment.** LPO levels in the brain samples were analyzed by a LPO assay kit according to manufacturer's instructions (Cayman's LPO Assay Kit – Catalog no. 705003). In brief, each brain sample was homogenized on ice in HPLC-grade water. The LPO was extracted from the homogenates into chloroform which helps to eliminate nearly all interfering substances from the sample. 500 µl of the chloroform extract of each sample was transferred to glass tubes. 450 µl of a chloroform-methanol solvent mixture and 50 µl of a freshly prepared chromogen were then added. The mixture was vortexed thoroughly and incubated at room temperature for 5 mins. The samples were transferred to a 96-well plate and absorbance at 500 nm was read using a plate reader. By substituting the corrected absorbance value for each sample into the equation obtained from the linear regression of the standard curve, the LPO concentrations of the samples were calculated.

**Western blot analysis.** Brains were homogenized on ice with an Ultra-Turrax (Ika, Werke) at 22,000 t/min in RIPA lysis buffer [consisting of 50mM Tris-HCl (pH 7.4), 150mM NaCl, 1% NP-40, 0.5% Sodium deoxycholate, 0.1% SDS, Boston BioProducts, Ashland, MA) supplemented with phosphatase and protease inhibitor cocktails (Roche Applied Science, Indianapolis, IN). Lysates were then resuspended in 4X LDS Sample Buffer (Invitrogen, Carlsbad, CA) and samples were directly loaded on a prechilled NuPage 4-12% Bis-Tris gradient gel (Invitrogen) and ran at slow voltage in the cold to preserve dimer forms of NOS. Fraction of the LDS resuspended lysates were reduced in Xt reducing agent (Bio-Rad, Hercules, CA) and boiled for 10 mins when used to detect total amounts of each NOS isoforms and their respective phosphorylated form. After transfer onto polyvinylidene difluoride membranes, blots were blocked for 1 h with 5% milk in Tris-buffered saline with 0.1% (vol/vol) Tween 20 (TBST) and then incubated with primary antibodies in 3% milk-TBST. β-Tubulin, total iNOS and nNOS antibodies were purchased from Santa Cruz Biotechnology (Santa Cruz, CA), total eNOS from Stressgen (KAP-NO002, Stressgen Bioregents, Victoria, BC, Canada), p-eNOS(S1176 and T495) from Cell Signaling Technology (Danvers, MA) and p-nNOS(S1417) from Millipore (Temecula, CA). Bound antibodies were detected with horseradish peroxydase-conjugated secondary antibodies (Cell Signaling Technology). Band intensity (mean optical density integrated for the band area) was quantified on unsaturated X-ray films scanned by a digital image analyzer (Quantity-One, Bio-Rad) and quantified with ImageJ.

**Calculation of *µ_app_* in microvessels. *µ_app_*** was estimated based on an empirical relationship given by Eq. (1) which is a function of systemic hematocrit (*H_sy_*_s_), vessel diameter (*D*) and plasma viscosity (*µ_p_*).

$\mu_{app}=\left[1+\left(\mu_{45}-1\right)\frac{\left(1-H_{sys}\right)^{C}-1}{\left(1-0.45\right)^{C}-1}\cdot\left(\frac{D}{D-1.1}\right)^{2}\right]\cdot\left(\frac{D}{D-1.1}\right)^{2}\cdot\mu_{p}$ (1)

where *µ_45_* (relative apparent blood viscosity for *H_sys_* = 0.45) and parameter *C* are derived as follow:

$\mu_{45}=6.\exp(-0.085D)+3.2-2.44\cdot\exp(-0.06D^{0.645})$ (2)

$C=(0.8+e^{-0.075D})\cdot(-1+\frac{1}{1+10^{-11}\cdot D^{12}})+\frac{1}{1+10^{-11}\cdot D^{12}}$ (3)

**Calculation of *γ_w_* in microvessels.** Under the assumption of a parabolic velocity profile in the blood vessel as described by Eq. (4), ***γ_w_*** was obtained by the gradient (derivative) of this profile at the vessel wall (Eq. (5)).

$V\left(r\right)=V_{c}\cdot(1-\left|\frac{2r}{D}\right|^{2})$ (4)

where *V(r)* refers to the velocity at radial position, *r*, in the vessel with *r* = 0 at the vessel centerline. *V_c_* is the centerline flow velocity and *D* is the vessel diameter.

$\gamma_{w}=\frac{dV\left(r=\frac{D}{2}\right)}{dr}=\frac{4V_{c}}{D}$ (5)

**Changes in *H_sys_*, *V_c_* and *D*.** Change in mean *H_sys_* from day 0 baseline in ECM = -17% (51% → 42%). In arterioles: change in mean *V_c_* from day 0 baseline in ECM (∆*V_c_*) = -48% (5.2mm/s → 2.7mm/s), corresponding change in mean *D* (∆*D*) = -21% (35µm → 28µm). In venules: ∆*V_c_* = -39% (3.8mm/s → 2.3mm/s), ∆*D* = -18% (35µm → 29µm).

**References**

1. Ong PK, Meays D, Frangos JA, Carvalho LJ (2012) A chronic scheme of cranial window preparation to study pial vascular reactivity in murine cerebral malaria. Microcirculation. In press.

2. Holtmaat A, Bonhoeffer T, Chow DK, Chuckowree J, De Paola V, et al. (2009) Long-term, high-resolution imaging in the mouse neocortex through a chronic cranial window. Nat Protoc 4: 1128-1144.

3. Cabrales P, Zanini GM, Meays D, Frangos JA, Carvalho LJ (2010) Murine cerebral malaria is associated with a vasospasm-like microcirculatory dysfunction, and survival upon rescue treatment is markedly increased by nimodipine. Am J Pathol 176: 1306-1315.

4. Qin X, Kwansa H, Bucci E, Dore S, Boehning D, et al. (2008) Role of heme oxygenase-2 in pial arteriolar response to acetylcholine in mice with and without transfusion of cell-free hemoglobin polymers. Am J Physiol Regul Integr Comp Physiol 295: R498-504.

5. Meng W, Ayata C, Waeber C, Huang PL, Moskowitz MA (1998) Neuronal NOS-cGMP-dependent ACh-induced relaxation in pial arterioles of endothelial NOS knockout mice. Am J Pathol 274: H411-415.

6. Ayata C, Moskowitz MA (2006) Cortical spreading depression confounds concentration-dependent pial arteriolar dilation during N-methyl-D-aspartate superfusion. Am J Physiol Heart Circ Physiol 290: H1837-1841.

7. Sun H, Patel KP, Mayhan WG (2001) Tetrahydrobiopterin, a cofactor for NOS, improves endothelial dysfunction during chronic alcohol consumption. Am J Physiol Heart Circ Physiol 281: H1863-1869.
